# Effectiveness and safety of poly (ADP-ribose) polymerase inhibitors in cancer therapy: A systematic review and meta-analysis

**DOI:** 10.18632/oncotarget.5367

**Published:** 2015-09-22

**Authors:** Zhengqiang Bao, Chao Cao, Xinwei Geng, Baoping Tian, Yanping Wu, Chao Zhang, Zhihua Chen, Wen Li, Huahao Shen, Songmin Ying

**Affiliations:** ^1^ Department of Respiratory and Critical Care Medicine, Second Affiliated Hospital, Institute of Respiratory Diseases, Zhejiang University School of Medicine, Hangzhou, China; ^2^ Department of Pharmacology, Zhejiang University School of Medicine, Hangzhou, China; ^3^ State Key Laboratory for Respiratory Diseases, Guangzhou, China

**Keywords:** clinical trials, PARP, BRCA, cancer, synthetic lethality, Olaparib

## Abstract

Poly (ADP-ribose) polymerase (PARP) inhibitors are a class of small-molecule drugs suppressing PARP enzymes activity, inducing the death of cells deficient in homologous recombination repair (HRR). HRR deficiency is common in tumor cells with BRCA gene mutation. Since their first clinical trial in 2003, PARP inhibitors have shown benefit in the treatment of HRR-deficient tumors. Recently, several randomized clinical trials (RCTs) have been conducted to investigate the potential benefit of administration of PARP inhibitors in cancer patients. However, the results remain controversial. To evaluate the efficiency and safety of PARP inhibitors in patients with cancer, we performed a comprehensive meta-analysis of RCTs. According to our study, PARP inhibitors could clearly improve progression-free survival (PFS), especially in patients with BRCA mutation. However, our study showed no significant difference in overall survival (OS) between the PARP inhibitors and controls, even in the BRCA mutation group. Little toxicity was reported in the rate of treatment correlated adverse events (AEs) in PARP inhibitor group compared with controls. In conclusion, PARP inhibitors do well in improving PFS with little toxicity, especially in patients with BRCA deficiency.

## INTRODUCTION

The poly (ADP-ribose) polymerases (PARPs) are a family of enzymes comprising 18 members, and they play a vital role in maintaining the stability of the genome [1]. Among them, PARP-1 and PARP-2 are best known for their contribution to DNA damage repair. PARP-1, which was first found ∼50 years ago, is activated by DNA damage and plays a crucial role in the repair of single-strand breaks [2]. Another family member, PARP-2, has 69% structural similarity to PARP-1, and some of their functions overlap [3]. Evidence demonstrates that PARP is significantly increased in some cancer types, compared with adjacent non-tumorous tissues [4, 5]. This suggests that inhibition of PARP may provide a novel strategy for cancer therapy.

Genomic instability accompanied with elevated DNA damage response is one of the most common features of human cancers [6], while Double strand breaks (DSBs) are the most severe type of DNA damage. DSBs are repaired mainly through non-homologous end-joining (NHEJ) and homologous recombination repair (HRR) pathways, which two play complementary roles [7]. PARP1-dependent end-joining (PARP-EJ) is a backup NHEJ repair pathway; when NHEJ is defective, PARP1-EJ pathway is activated [8, 9]. PARP inhibitors (such as Olaparib, Iniparib, Veliparib, Rucaparib, and Niraparib), a class of small-molecule drugs inhibiting PARP enzymes, can induce synthetic lethality in HRR deficiency cancer cells [10]. HRR deficiency is common in tumor cells, for example with BRCA gene mutations. *In vitro* experiments have established that cells with defective HRR are killed by PARP inhibitors [11]. Since their first clinical trial in 2003 [12], PARP inhibitors have shown benefit in the treatment of HRR-deficient tumors. Many kinds of PARP inhibitors have been designed since then; for example, Olaparib has been clinically approved for use in human testing by the Food and Drug Administration (FDA) in the USA.

However, on the other hand, serious adverse events have been reported in the PARP inhibitor arms of some clinical trials, and the therapeutic effect also seemed to be unsatisfactory [13, 14]. Since their appearance, PARP inhibitors have attracted controversy as to whether they are effective and safe anti-tumor agents. Thus, we set out to make a systematic review and meta-analysis of randomized controlled trials (RCTs) to gain insight relative risks and benefits of PARP inhibitors in patients with cancer.

## RESULTS

### Literature search

**Figure 1:**
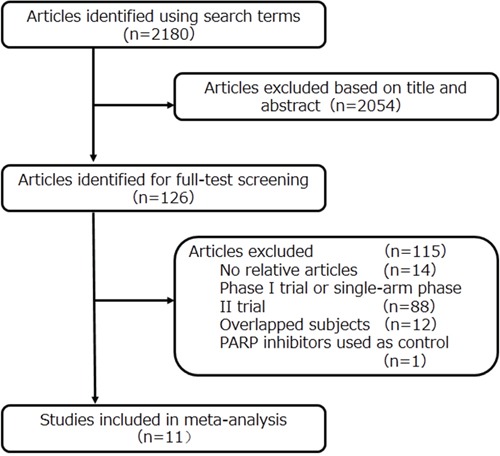
Flow diagram of the literature search and trial selection process

We initially identified 2180 potentially eligible studies by title and abstract screening. However, 2054 were excluded as they were not relevant to our analysis, leaving 126 articles for full review. After assessing the full texts of these potentially relevant studies, 115 were excluded for the following reasons: 14 contained no relative outcomes; 88 were phase I or single-arm phase II trials; 12 were duplicate publication; and 1 used PARP inhibitors in both the experimental and control groups. Ultimately, 11 eligible RCTs [15–25] involving a total of 2274 patients were included for analyses. A flow diagram of the trial selection process is shown in Figure 1. One article (OZA, 2013) [25] from EMBASE was partly overlapped with a previous publication (OZA 2015) [15], but it provided elaborated progression-free survival (PFS) data on the BRCA status, which was not mentioned in previous article, so it was also included.

Of all 11 RCTs, 4 were BRCA mutation-correlated; 6 used the PARP inhibitor Olaparib, 4 used Iniparib, and 1 used Veliparib; 4 were about ovarian cancer, 2 about lung cancer, 2 about breast cancer, 1 about gastric cancer, 1 about pancreatic cancer, and 1 about ovarian, peritoneal, and fallopian tube cancers. The characteristics of 11 included trials are presented in Table 1.

**Table 1:** Characteristics of the trials included in the meta-analysis

Source	Phase	Histology	Treatment arm	No.of patients Enrolled	Female (%)	Age in years, Median (range)	BRCA Mutation Patients	Median OS (months)	Median PFS (months)
Oza, 2015^12^	II	Ovarian cancer	Olaparib (200mg) + paclitaxel/carboplatin, Olaparib (400mg);	81	100	59.0 (27–78)	20	33.8	12.2
			Paclitaxel/carboplatin	81		62.0 (31–79)	21	37.6	9.6
Kummar, 2015^16^	II	Ovarian cancer, and others	Veliparib (60 mg) +cyclophosphamide;	37	NA	NA	NA	NA	2.1
			Cyclophosphamide	38		NA	NA	NA	2.3
Novello, 2014^17^	II	Lung cancer	Iniparib (5.6 mg/kg) +Gemcitabine/Cisplatin;	80	24.4	59.0 (37–73)	NA	12	5.7
			Gemcitabine/Cisplatin	39		58.0 (29–73)	NA	8.5	4.3
Ledermann, 2014^18^	II	Ovarian cancer	Olaparib 400 mg (BRCA mutation);	74	100	57.5 (38–89)	74	34.9	11.2
			Placebo (BRCA mutation);	62		55.0 (33–84)	62	31.9	4.3
			Olaparib 400mg (BRCA wild-type);	57		62.0 (21–80)	0	24.5	7.4
			Placebo (BRCA wild-type)	61		63.0 (49–79)	0	26.2	5.5
Kaye, 2012^19^	II	Ovarian cancer	Olaparib (200 mg);	32	100	58.5 (45–77)	32	NA	6.5
			Olaparib (400 mg);	32		53.5 (35–76)	32	NA	8.8
			Placebo	33		53.0 (43–81)	33	NA	7.1
O'Shaughnessy, 2011^20^	II	Breast cancer	Iniparib (5.6 mg/kg) +Gemcitabine/Carboplatin;	61	100	56.0 (34–76)	NA	12.3	5.9
			Gemcitabine/Carboplatin	62		53.0 (26–80)	NA	7.7	3.6
Spigel, 2013^21^	III	Lung cancer	Iniparib (5.6 mg/kg) +Gemcitabine/Carboplatin;	390	NA	Total: 66.0 (21–86)	NA	8.9	4.8
			Gemcitabine/Carboplatin	390			NA	8.9	4.9
Bang, 2013^22^	II	Gastric cancer	Olaparib (100 mg) + paclitaxel, Olaparib(200mg);	61	NA	NA	NA	NA	NA
			Placebo +paclitaxel, Placebo	62		NA	NA	NA	NA
O'Shaughnessy, 2014^23^	III	Breast cancer	Iniparib (5.6 mg/kg) +Gemcitabine/Carboplatin;	261	100	53.0 (NA)	NA	11.8	5.1
			Gemcitabine/Carboplatin	258		54.0 (NA)	NA	11.1	4.1
Bendell, 2015^24^	I	Pancreatic cancer	Olaparib (100 mg) +gemcitabine;	15	50	65.0 (47–79	3	NA	NA
			Gemcitabine	7		66.0 (44–73)	0	NA	NA
Oza, 2013^25^	II	Ovarian cancer	Olaparib (200 mg) +paclitaxel/carboplatin, Olaparib (400 mg);	81	100	59.0 (27–78)	NA	NA	NA
			Paclitaxel/carboplatin	81		62.0(31–79)	NA	NA	NA

NA: Not available; PFS: Progression-free survival; OS:Overall survival.

### Outcomes

#### Progression-free survival

**Figure 2:**
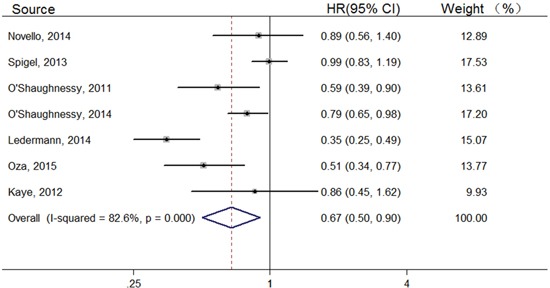
Forest plots of the pooled HRs for PFS by overall population

7 trials reported PFS of the overall population. Overall, PFS was significantly longer in the PARP inhibitors group than in the control group [Hazard ratio (HR), 0.67; 95% confidence interval (CI), 0.50–0.90] (Figure 2). BRCA mutation status was known in 3 trials, and pooled results showed a HR of 0.32 (95% CI, 0.11–0.94); 6 trials on BRCA status unknown or non-mutation subgroup, the HR was 0.78 (95% CI, 0.65–0.95) (Table 2). 4 trials provided data on Iniparib and 3 trials on Olaparib. For Iniparib, no significant difference was observed between experiment group and control group (HR, 0.83; 95%CI, 0.68–1.02) (Table 2). For Olaparib, a significant improvement in PFS was recorded in the Olaparib group compared with the control group (HR, 0.50; 95% CI, 0.32–0.80) (Table 2). In the subgroup analysis by cancer type, 2 trials concerning lung cancer, and they gave a HR of 0.98 (95% CI, 0.83–1.15); 3 trials on ovarian cancer, the HR of PFS was 0.50 (95%CI, 0.32–0.80); 2 trials about breast cancer, and they gave a HR of 0.72 (95% CI, 0.56–0.94) (Table 2).

**Table 2:** Summary results of the pooled HRs for PFS and OS by subgroup analysis

		Pooled PFS				Pooled OS			
		No.of trials	HR (95%CI)	*I^2^*	*P*	No.of trials	HR (95%CI)	*I^2^*	*P*
BRCA status									
	BRCA status unknown or non-mutation	6^17,18,20,21,23,25^	0.78(0.65,0.95)	49.90%	0.076	5^17,18,20,21,23^	0.89(0.73,1.09)	50.80%	0.087
	BRCA mutation	3^15,18,19^	0.32(0.11,0.94)	85.60%	0.001	3^15,18,19^	0.83(0.57,1.23)	0.00%	0.604
Drug type									
	Iniparib	4^17,20,21,23^	0.83(0.68,1.02)	51.10%	0.105	4^17,20,21,23^	0.86(0.67,1.10)	63.00%	0.044
	Olaparib	3^15,18,19^	0.50(0.32,0.80)	68.90%	0.040	3^15,18,19^	0.99(0.78,1.25)	0.00%	0.542
Cancer type									
	Ovarican cancer	3^15,18,19^	0.50(0.32,0.80)	68.90%	0.040	3^15,18,19^	0.99(0.78,1.25)	0.00%	0.542
	Breast cancer	2^20,23^	0.72(0.56,0.94)	33.70%	0.219	2^20,23^	0.74(0.49,1.12)	62.90%	0.100
	Lung cancer	2^17,21^	0.98(0.83,1.15)	0.00%	0.672	2^17,21^	1.00(0.76,1.31)	35.10%	0.214

PFS: Progression-free survival; OS:Overall survival; *P: P*-value of Q-test for heterogeneity test.

#### Overall survival rates

**Figure 3:**
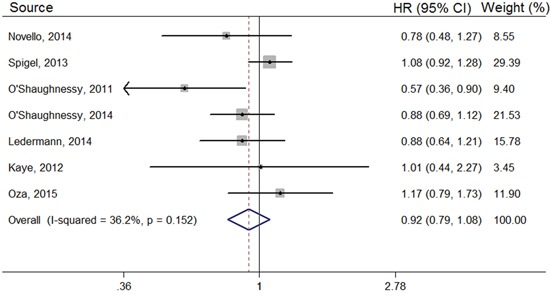
Forest plots of the pooled HRs for OS by overall population

Our meta-analysis showed no significant difference in overall survival rates between the PARP inhibitor and placebo arms in the overall population (HR, 0.92; 95% CI, 0.79–1.08) (Figure 3). In the BRCA mutation group, the HR was 0.83 (95% CI, 0.57–1.23); and in the BRCA status unknown or non-mutation subgroup, the HR was 0.89 (95% CI, 0.73–1.09) (Table 2). Olaparib showed no statistical difference in overall survival rates, with an HR of 0.99 (95% CI, 0.78–1.25), compared with controls group; Similar results was observed in the Iniparib group (HR, 0.86; 95% CI, 0.67–1.10) (Table 2). In addition, compared with control group, PARP inhibitors showed no statistically significant difference in improving the overall survival (OS) of patients with ovarian (HR, 0.99; 95% CI, 0.78–1.25), breast (HR, 0.74; 95% CI, 0.49–1.12), or lung cancer (HR, 1.00; 95% CI, 0.76–1.31) (Table 2).

### Safety

To evaluate the safety of PARP inhibitors, we analyzed the risk factor of any side-effects in the overall population with Grade 3 (G3) or more serious adverse events (AEs). Furthermore, since two PARP inhibitors were mainly used in the RCTs, we also evaluated the safety of the Iniparib and Olaparib subgroups separately.

Analysis of patients in the overall population showed that, compared with the control arms, PARP inhibitors were associated with a decreased risk of asthenia (RR, 0.34; 95%CI, 0.14–0.82) but increased risk of neutropenia (RR, 1.14; 95%CI, 1.01–1.29). In our study, we did not find any association between PARP inhibitors and other AEs (Table 3). In addtion, there were no significant differences in the AEs in the Olaparib or Iniparib subgroup (Supplementary Tables 1 and 2).

**Table 3:** Relative risks with 95% confidence intervals for common adverse events (Grade ≥ 3)

Adverse event	No.of Trials	Subjects	RR [95% CI]	*P*	*I^2^ (%)*	*P^b^*
Abdominal pain	5	350/265	0.48[0.18,1.25]	0.13	0.00	0.86
Anaemia	8	703/609	1.45[0.77,2.75]	0.25	49.00	0.06
Anorexia	2	72/66	0.67[0.11,4.17]	0.67	0.00	0.61
Arthralgia	2	193/187	2.96[0.31,28.17]	0.34	0.00	0.97
Asthenia	3	278/199	0.34[0.14,0.82]	0.02	2.00	0.36
Constipation	4	454/374	1.63[0.46,5.81]	0.45	0.00	0.98
Cough	2	312/303	1.32[0.25,6.97]	0.74	0.00	0.54
Dehydration	2	94/97	3.09[0.33,29.18]	0.32	0.00	1.00
Diarrhoea	5	590/502	1.90[0.84,4.29]	0.12	0.00	0.80
Dyspnea	4	405/349	1.14[0.53,2.46]	0.74	16.00	0.31
Fatigue	5	548/450	1.34[0.82,2.19]	0.24	0.00	0.62
Headache	2	391/372	1.45[0.41,5.20]	0.57	8.00	0.30
Increased ALT	3	327/310	0.95[0.25,3.62]	0.94	42.00	0.18
Increased AST	2	312/303	1.10[0.56,2.17]	0.78	0.00	0.52
Leukopenia	4	427/380	0.99[0.71,1.38]	0.95	0.00	0.44
Nausea	6	605/509	1.17[0.51,2.66]	0.71	10.00	0.35
Neutropenia	7	639/577	1.14[1.01,1.29]	0.03	0.00	0.46
Peripheral edema	3	327/310	1.10[0.28,4.42]	0.89	0.00	0.73
Pulmonary embolism	2	93/46	1.02[0.29,3.54]	0.98	0.00	0.50
Thrombocytopenia	4	442/387	1.26[0.99,1.60]	0.06	0.00	0.88
Vomiting	6	605/509	1.43[0.66,3.09]	0.36	0.00	0.89

RR: Relative risk; CI: Confidence interval; *P^b^*: *P*-value of Q-test for heterogeneity test.

### Analysis of publication bias

We used Funnel plot and Egger's regression asymmetry test to access the publication bias of literatures. Arrangement of data points did not reveal any evidence of obvious asymmetry. This was further confirmed by Egger's linear regression asymmetry test for each outcome and the results still did not show any evidence of publication bias (PFS: *t* = −1.24, *P* = 0.271; OS: *t* = −1.30, *P* = 0.251) (Supplementary Figures 1 and 2).

## DISCUSSION

To our best knowledge, this study is the first systematic review and meta-analysis to evaluate the efficiency and safety of the novel antitumor PARP inhibitors. In our study, all RCTs included were published from 2011 to 2015, which reflects the popularity of PARP inhibitors in the past few years. Of all the 2274 patients, several types of cancers were reported, such as ovarian, lung, breast, and gastric cancers. Besides these, many trials [26–32] in different kinds of cancers not eligible for inclusion are still under way. Although the results have not come out, it is possible that PARP inhibitors may work in patients against some certain types of tumors. Various inhibitors suppressing PARP enzymes were involved in this study, including Olaparib, Iniparib, and Veliparib. It is worth noting that Olaparib was recently approved for use in human testing by the FDA in 2014 [33]. Will PARP inhibitors be a powerful and safe strategy for personalized cancer treatment in the future? According to our study, PARP inhibitors do well in prolonging the PFS of cancer patients, despite of some reported adverse events.

In our study, PARP inhibitors significantly improved the PFS in the overall population (HR, 0.67; 95% CI, 0.50–0.90), while the overall HR of the BRCA status unknown or non-mutation group was a little higher (HR, 0.78; 95% CI, 0.65–0.95). However, the difference was greater in the BRCA mutation group (HR, 0.32; 95% CI, 0.11–0.94). There is no doubt that BRCA mutation made a major contribution to the improved results. Individuals with BRCA mutation are at an increased risk of developing breast, ovarian, and other cancers [34]. More than 1 million women develop breast or ovarian cancer every year worldwide, and about 10% of them have a BRCA mutation [35–37], moreover that cancer patients with BRCA mutation have better outcomes than non-BRCA carriers [38, 39]. And a recent study [40] suggested that BRCA mutation should be taken into account when devising therapeutic strategies. BRCA plays a vital role in DNA damage repair by the HRR process, while PARP enzymes are involved in crucial complementary repair process [41]. With BRCA mutation, cancer cells are unable to perform HRR efficiently [42], then PARP in turn plays a major role in repairing damaged DNA to maintain cell survival. Thus, PARP could be targeted for treating BRCA-mutant tumors using a synthetic lethal approach. In this analysis, PARP inhibitors appeared to be efficient in killing BRCA-deficient cancer cells and prolonging the PFS of patients, mainly due to suppression of PARP enzyme activity [43].

Since PARP inhibitors have emerged as promising antitumor drugs, many efforts have been made to develop compounds such as Olaparib, Iniparib, Veliparib, and others as antineoplastic agents [44]. In our study, the main PARP inhibitors included were Olaparib and Iniparib. Olaparib has already shown benefit in treating patients with BRCA mutation, and our results confirmed that again, with an overall HR of 0.50 (95% CI, 0.32–0.80) between the Olaparib and control arms. Although Iniparib had a tendency to improve the PFS, the difference did not reach a statistically significant. The mechanism of action of Iniparib seems not to be closely correlated to PARP enzymes, it was reported that Iniparib carries a carboxyl group swiveled around an amino bond, and this may weakens its ability to bind PARP [45]. Although in the four trials with Iniparib it was combined with gemcitabine, cisplatin, or carboplatin, data [46] have already revealed that Iniparib fails to sensitize cells to cisplatin, gemcitabine, or paclitaxel. We suppose that one possibility of the difference, caused between the Iniparib and Olaparib subgroups, may be different criteria of patient enrolment. Patients enrolled in the Olaparib trials mostly had BRCA mutations, while those in the Iniparib subgroup mainly had non-mutation BRCA status. It should also be noted that an initial submission of Olaparib to FDA was rejected. The application was only accepted following addition data provided to support that benefit was restricted to the BRCA mutated patients.

Soon after their development, PARP inhibitors were used in hundreds of clinical trials, and many different kinds of tumors with or without BRCA mutation were involved [47]. For example, Olaparib was reported to be effective against several tumor types including ovarian, breast, pancreatic, and prostate cancers [48]. The main tumor types included in our study were ovarian, breast, and lung cancers. According to our results, there was significant statistical heterogeneity in the PFS of the ovarian cancer subgroup (HR, 0.50; 95%CI, 0.32–0.80), while in the lung (HR, 0.98; 95%CI, 0.83–1.15) and breast cancer groups (HR, 0.72; 95%CI, 0.56–0.94), there seemed to be no odds difference between the PARP inhibitor and control arms. As far as we know, various PARP inhibitors were designed to suppress tumors with BRCA mutation, no matter what kind of cancer. Thus, the reason may be that most of the patients in the ovarian cancer group had BRCA mutation, while those in lung and breast cancer subgroups did not. The results would be better if the patients in lung and breast cancer group were also with BRCA mutation as those in ovarian cancer group.

Consistent with previous hypothesis, our study show that cancer patients with BRCA mutation may increase sensitivity to PARP inhibitors [49]. PARP inhibitors can induce synthetic lethality in HRR deficient cancer cells, such as BRCA dysfunction [50]. BRCA mutation was at a high prevalence among breast cancer patients. About 20% of breast cancer patients were BRCA mutations carriers [51, 52]. Beyond breast cancer, several other malignancies, including ovarian cancer, pancreatic cancer, melanoma, and prostate cancer, were also correlated to BRCA mutations [53]. On top of BRCA, mutations of other DNA repair genes, such as RAD51, ATM, PALB2, CHEK2, may also increase sensitivity to PARP inhibitors [54]. It is therefore important to perform genetic testing and in prior to this therapy.

However, PAPR inhibitors failed to improve the OS of cancer patients in this analysis. Apart from the overall level, we also analyzed the OS from the perspective of cancer type, PARP inhibitor category, and BRCA status. According to our analysis, other than the OS of the Iniparib subgroup, none of the results showed significant differences between the PARP inhibitor and control arms. And even the result in the Iniparib subgroup was not satisfactory, with an HR of 0.86 (95%CI, 0.67–1.10). Although PARP inhibitors did not statistically improve the OS, in some individual trials, they were reported to clearly increase it. For example, Novello *et al*^17^ reported that the median OS in the PARP inhibitor arm was 12 months compared with 8.5 months in the control arm; and in another trial^20^, the OS was prolonged from 7.7 months to 12.3 months by PARP inhibitors. Research on PARP inhibitors is still ongoing, and many aspects need further improvement. Also, the inherent relationship between PFS and OS should be taken into account, since PFS is contained within OS. PARP inhibitors may be able to effectively improve the PFS, but this was not strong enough to translate PFS effects into OS improvement.

Today, many traditional anti-cancer drugs are able to kill tumor cells, but their toxicity to normal cells restricts their clinical application. PARP inhibitors suppress DNA repair, and kill cancer cells through “synthetic lethality” [55]. In this way, PARP inhibitors are applicable across various cancers, improving the efficacy and reducing the toxicity of individualized therapies. Our study revealed the benefit of the low toxicity of PARP inhibitors. Compared with the control arms, no treatment-correlated risks were seen in the Iniparib and Olaparib subgroups. Only a slightly decreased risk of asthenia (RR, 0.34; 95% CI, 0.14–0.82) and increased risk of neutropenia (RR, 1.14; 95% CI, 1.01–1.29) were seen in the overall population, suggesting that the PARP inhibitors are well-tolerated.

In conclusion, based on the available observational studies, PARP inhibitors do better in improving PFS with little toxicity, especially in patients with BRCA deficiency. However, they fail to increase the OS.

## MATERIALS AND METHODS

### Study selection

We carried out a comprehensive search to identify potential articles in PUBMED and EMBASE up to January 2015, using the search terms: “PARP inhibitors” or “Olaparib” or “Iniparib” or “Veliparib” or “Rucaparib” or “Niraparib” or “Talazoparib” and “cancer” or “tumor” or “carcinoma”, limited to clinical trials. There was no limit on the language of publication. In order to ensure the completeness and quality of the results, relevant scientific meetings were retrieved, and unpublished trials were checked in the clinical trial registry (http://www.clinicaltrials.gov).

To be included, studies had to be RCTs and had to report at least one outcome of interest, such as PFS, OS, and AEs. Single-arm trials and trials in which PARP inhibitors were used in both arms were excluded, on account of the absence of control groups. Trials in which PARP inhibitors were used to treat other diseases were also excluded. In all the included RCTs, PARP inhibitors were used alone or combination with other chemotherapeutic agents as the treatment group, while in the control group placebo or other chemotherapeutic agents were used. Two investigators reviewed the articles independently to exclude irrelevant and overlapping studies.

### Data extraction

We collected the following information from all the included RCTs: first author's surname, year of publication, number of participants, histology, trial phase, treatment arm, median age, BRCA status, median OS, and median PFS. In addition, the HR of the median OS and median PFS with 95% CIs were extracted from most of the trials to evaluate the curative effect of PARP inhibitors. Information on AEs was also retrieved to calculate the safety of PARP inhibitors.

### Statistical analysis

All analyses were done with Stata version 12 (StataCorp, College Station, Texas) and Review Manager (version 5.1, The Cochrane Collaboration, Oxford, UK). A 2-tailed *P* value of less than 0.05 was judged as statistically significant. HR and 95% CI were used to assess the OS and PFS between PARP inhibitors group and control group. In addition, we extracted dichotomous data form all studies reporting number of patients with adverse events and total participants and pooled them to calculate RR with 95% CI. The degree of heterogeneity was measured by the *I^2^* statistic, with *I*^2^ < 25%, 25–75% and > 75% to represent low, moderate and high degree of inconsistency, respectively [56]. Statistical heterogeneity was defined as an *I*^2^ statistic value of more than 50% [56]. In analyses, if the heterogeneity was low then we used a fixed-effect model, or else applied the random-effect model. We further performed a subgroup analysis by the status of BRCA, tumor type, and different kinds of PARP inhibitors (Iniparib and Olaparib). Funnel plot and Egger's regression asymmetry test were used to access the publication bias of literatures [57].

## SUPPLEMENTARY FIGURES AND TABLES


