# Supplementary material for: Effectiveness and safety of poly (ADP-ribose) polymerase inhibitors in cancer therapy: A systematic review and meta-analysis
Source: Oncotarget. 2015 Sep 22;7(7):7629–39. doi: 10.18632/oncotarget.5367 (PMC4884943; doi:10.18632/oncotarget.5367)
Supplement: Supplementary file 1 [file oncotarget-07-7629-s001.pdf]

SUPPLEMENTARY FIGURES AND TABLES

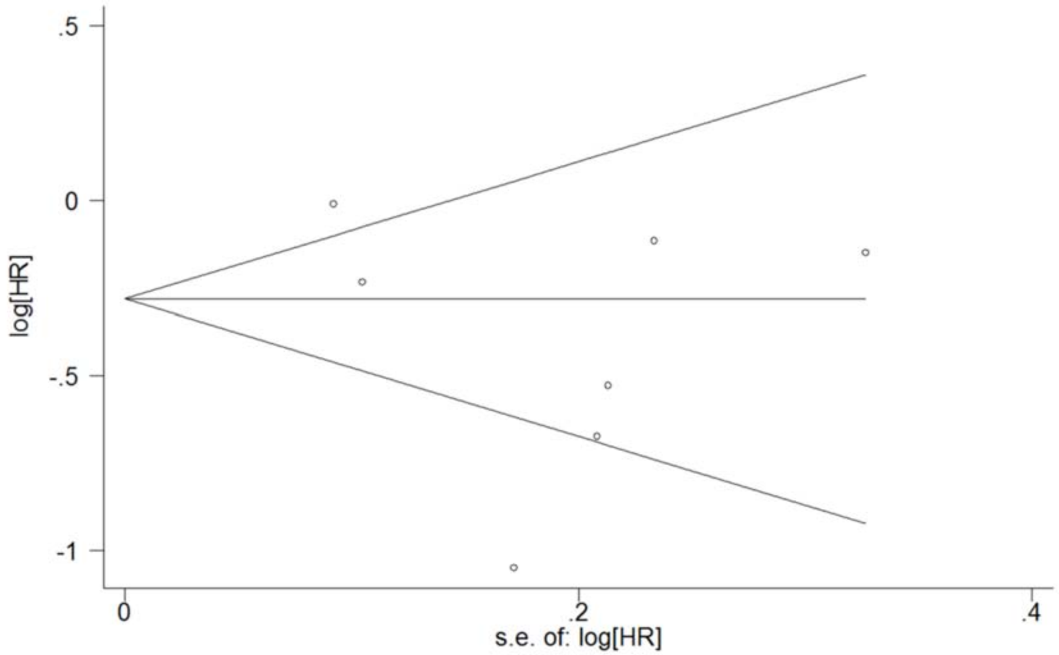

Supplementary Figure S1: Begg's funnel plot of the 7 eligible RCTs assessing PFS of cancer patients.

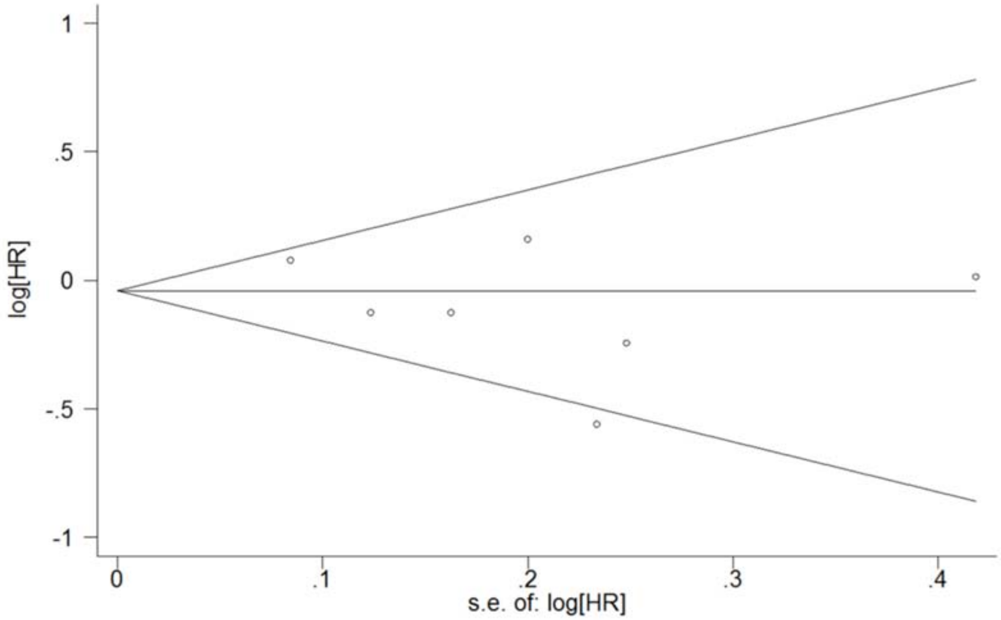

Supplementary Figure S2: Begg's funnel plot of the 7 eligible RCTs assessing OS of cancer patients.

Supplementary Table S1: Relative risks with 95% confidence intervals for common adverse events (Grade ≥ 3) in Iniparib subgroup

Adverse event	No.of trials	Subjects	RR[95% CI]	*P*	I^2 (%)	P^b
Abdominal pain	2	135/98	0.53[0.06,4.71]	0.57	0.00	0.37
Anaemia	2	135/98	1.50[0.80,2.79]	0.20	0.00	1.00
Constipation	2	135/98	1.29[0.23,7.31]	0.77	0.00	0.84
Diarrhoea	2	135/98	1.22[0.15,9.75]	0.85	0.00	0.86
Dyspnea	2	135/98	0.54[0.17,1.72]	0.30	0.00	0.41
Leukopenia	2	135/98	0.80[0.44,1.45]	0.46	0.00	0.33
Nausea	2	135/98	1.83[0.13,26.33]	0.66	52.00	0.15
Neutropenia	2	135/98	1.00[0.80,1.27]	0.97	0.00	0.40
Thrombocytopenia	2	135/98	1.27[0.82,1.97]	0.28	0.00	0.69
Vomiting	2	135/98	1.01[0.30,3.34]	0.99	0.00	0.98

RR: Relative risk; CI: Confidence interval; P^b: *P*-value of Q-test for heterogeneity test.

Supplementary Table S2: Relative risks with 95% confidence intervals for common adverse events (Grade≥3) in Olaparib subgroup.

Adverse event	No.of trials	Subjects	RR[95% CI]	*P*	I^2(%)	P^b
Abdominal pain	3	215/167	0.47[0.16,1.36]	0.17	0.00	0.78
Anaemia	4	276/229	1.79[0.53,6.07]	0.35	37.00	0.19
Asthenia	2	200/160	1.04[0.13,8.33]	0.97	0.00	0.42
Diarrhoea	2	200/160	1.76[0.57,5.51]	0.33	13.00	0.28
Fatigue	3	215/167	1.45[0.62,3.42]	0.39	0.00	0.40
Nausea	3	215/167	3.35[0.60,18.82]	0.17	0.00	0.79
Neutropenia	3	212/197	1.37[0.81,2.32]	0.23	16.00	0.30
Vomiting	3	215/167	1.43[0.31,6.49]	0.64	0.00	0.63

RR: Relative risk; CI: Confidence interval; P^b: *P*-value of Q-test for heterogeneity test.
